# P-545. Feasibility, Fidelity, and Effectiveness of Administering CABENUVA in Infusion Centers

**DOI:** 10.1093/ofid/ofae631.744

**Published:** 2025-01-29

**Authors:** Cassidy Gutner, Conn M Harrington, Gilda Bontempo, Sivakumar Pazhamalai, Abhishek Kumar, Kelly Rimler, Karen Davis, Will Williams, Deanna Merrill, Lisa Petty, Cindy Garris, Supriya Sarkar, Katheryne Downes, Patrick Daniele, Bridget Gaglio, Katie May, Maggie Czarnogorski

**Affiliations:** ViiV Healthcare, Durham, North Carolina; ViiV Healthcare, Durham, North Carolina; ViiV Healthcare, Durham, North Carolina; GSK, Bangalore, Karnataka, India; GSK, Bangalore, Karnataka, India; GSK, Bangalore, Karnataka, India; ViiV Healthcare, Durham, North Carolina; GlaxoSmithKline, Collegeville, Pennsylvania; ViiV Healthcare, Durham, North Carolina; ViiV Healthcare, Durham, North Carolina; ViiV Healthcare, Durham, North Carolina; ViiV Healthcare, Durham, North Carolina; Evidera, Bethesda, Maryland; Evidera, Bethesda, Maryland; Evidera, Bethesda, Maryland; Infusion Associates, Grand Rapids, Michigan; ViiV Healthcare, Durham, North Carolina

## Abstract

**Background:**

Administering intramuscular CABENUVA at infusion centers (ICs) offers a convenient alternative for patients to receive treatment. Giving Long Acting CABENUVA in Infusion centERs (GLACIER) examined feasibility and fidelity of delivering monthly and every 2-monthly CABENUVA at ICs from the perspective of patient study participants (PSP) over 8 months.

**Figure:**
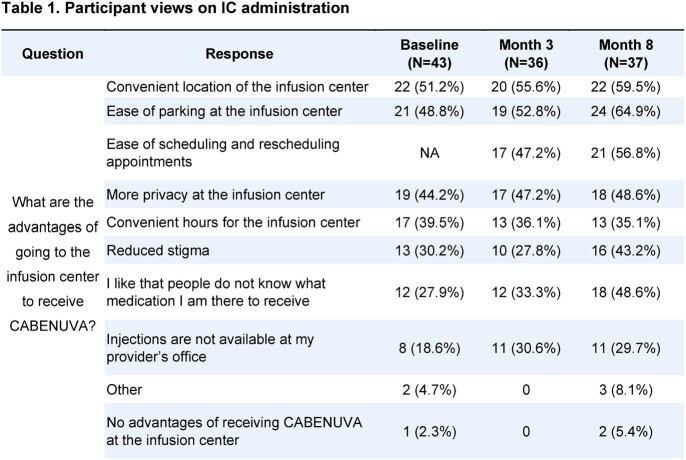

**Methods:**

Study data includes clinical data and questionnaires to examine PSPs experiences receiving CABENUVA in IC routine care. Quantitative questionnaires include the Feasibility of Intervention Measure (FIM), the Acceptability of Intervention Measure (AIM), and implementation questions. Qualitative interviews were completed with a subset of PSPs.

**Results:**

Enrolled participants (n=44) had a mean age of 46.8, 20.5% female at birth, and 51.2% had not previously received CABENUVA. 96.4% (187/194) of injections were within the treatment window. No study withdrawal or treatment discontinuation were due to AEs. There were 16 ISR AEs in 12 participants and 19 treatment related AE in 13 participants; most were mild (Grade 1) or moderate (Grade 2). Injection site discomfort (14%) and pain (14%) were cited most frequently. No SAEs were reported. At Month 8, IC administration was highly feasible (FIM: *M*=4.41) and acceptable (AIM: *M*=4.51). Advantages of ICs included ease of parking, convenient location, ease of scheduling/rescheduling, privacy, others not knowing what medication they received, and reduced stigma (Table 1). At Month 8, 94.6% of PSPs reported being very or extremely satisfied with the ICs care. Qualitative interviews highlighted positive views of IC administration, including staff relationships and continuity in care as key acceptability factors.

**Conclusion:**

CABENUVA administration at ICs was safe, effective, and convenient. High levels of adherence to the treatment window, acceptability, and feasibility were reported. Rapport with IC staff, continuity in care, reduction in logistical barriers and the perception of decreased stigma highlight that ICs can be a valuable alternative site of care for CAEBNUVA administration. ICs should be considered to improve convenience of treatment administration and when HCP offices have limited capacity.

**Disclosures:**

**Cassidy Gutner, PhD**, GSK: Stocks/Bonds (Public Company)|ViiV Healthcare: Employee **Conn M. Harrington, BA**, GSK: Stocks/Bonds (Public Company)|ViiV Healthcare: Employee **Gilda Bontempo, MD**, GSK: Stocks/Bonds (Public Company)|ViiV Healthcare: Employee **Sivakumar Pazhamalai, BPharm**, GSK: Employee **Abhishek Kumar, MSc**, GSK: Employee|GSK: Stocks/Bonds (Public Company) **Kelly Rimler, MS**, GSK: Employee **Karen Davis, MS, RN**, GSK: Stocks/Bonds (Public Company)|ViiV Healthcare: Employee **Will Williams, n/a**, GSK: Employee|GSK: Stocks/Bonds (Public Company) **Deanna Merrill, PharmD, MBA, AAHIVP**, GSK: Stocks/Bonds (Public Company)|ViiV Healthcare: Employee **Lisa Petty, MT(ASCP)**, GSK: Stocks/Bonds (Public Company)|ViiV Healthcare: Employee **Cindy Garris, MS**, GSK: Stocks/Bonds (Public Company)|ViiV Healthcare: Employee **Supriya Sarkar, PhD, MPH**, GSK: Stocks/Bonds (Public Company)|ViiV Healthcare: Full time employee **Patrick Daniele, MS**, GSK: Advisor/Consultant|ViiV Healthcare: Advisor/Consultant **Bridget Gaglio, PhD, MPH**, Thermo Fisher Scientific: Stocks/Bonds (Public Company) **Maggie Czarnogorski, MD MPH**, GSK: Stocks/Bonds (Public Company)|ViiV Healthcare: Employee

